# GABAergic feedback signaling into the calyces of the mushroom bodies enables olfactory reversal learning in honey bees

**DOI:** 10.3389/fnbeh.2015.00198

**Published:** 2015-07-29

**Authors:** Constance Boitard, Jean-Marc Devaud, Guillaume Isabel, Martin Giurfa

**Affiliations:** ^1^Research Center on Animal Cognition (UMR 5169), Centre National de la Recherche Scientifique (CNRS)Toulouse, France; ^2^Research Center on Animal Cognition (UMR 5169), Université Paul SabatierToulouse, France

**Keywords:** reversal learning, honey bees, mushroom bodies, GABAergic signaling

## Abstract

In reversal learning, subjects first learn to respond to a reinforced stimulus A and not to a non-reinforced stimulus B (A^+^ vs. B^−^) and then have to learn the opposite when stimulus contingencies are reversed (A^−^ vs. B^+^). This change in stimulus valence generates a transitory ambiguity at the level of stimulus outcome that needs to be overcome to solve the second discrimination. Honey bees (*Apis mellifera*) efficiently master reversal learning in the olfactory domain. The mushroom bodies (MBs), higher-order structures of the insect brain, are required to solve this task. Here we aimed at uncovering the neural circuits facilitating reversal learning in honey bees. We trained bees using the olfactory conditioning of the proboscis extension reflex (PER) coupled with localized pharmacological inhibition of Gamma-AminoButyric Acid (GABA)ergic signaling in the MBs. We show that inhibition of ionotropic but not metabotropic GABAergic signaling into the MB calyces impairs reversal learning, but leaves intact the capacity to perform two consecutive elemental olfactory discriminations with ambiguity of stimulus valence. On the contrary, inhibition of ionotropic GABAergic signaling into the MB lobes had no effect on reversal learning. Our results are thus consistent with a specific requirement of the feedback neurons (FNs) providing ionotropic GABAergic signaling from the MB lobes to the calyces for counteracting ambiguity of stimulus valence in reversal learning.

## Introduction

Associative learning may be divided in two main categories: (i) elemental learning, in which linear and unambiguous links are established between events (Rescorla and Wagner, 1972); and (ii) non-elemental learning, in which the links established between events are ambiguous and non-linear (Rudy and Sutherland, 1989; Sutherland and Rudy, 1989). In Pavlovian learning, for instance, two levels of stimulus ambiguity are represented by differential and reversal conditioning. In differential conditioning, two conditioned stimuli (CS) A and B are unambiguously associated with an unconditioned stimulus (US) and with the absence of US, respectively (A^+^ vs. B^−^). In reversal conditioning, a first differential conditioning phase (A^+^ vs. B^−^) is followed by a second phase, in which stimulus contingencies are reversed (A^−^ vs. B^+^). Thus, the addition of the second phase generates a transient ambiguity of stimulus outcome (A^+^ → A^−^ and B^−^ → B^+^) that needs to be overcome.

In mammals, different brain structures are associated with learning forms exhibiting different levels of ambiguity. While the hippocampus seems to be dispensable for non-ambiguous, elemental discriminations (Rudy and Sutherland, 1989; Sutherland and Rudy, 1989; Stupien et al., 2003) it is required—together with the cortical system—for learning of non-linear, ambiguous discriminations (Rudy and Sutherland, 1989; Sutherland and Rudy, 1989; Dusek and Eichenbaum, 1997; Rudy and O’Reilly, 1999, 2001; Rudy et al., 2002; Stupien et al., 2003). In insects, comparable results were found in the honey bee, an insect which has a model status for studies on learning and memory (Menzel, 1999; Giurfa, 2003, 2007; Giurfa and Sandoz, 2012), and which learns efficiently olfactory reversal discriminations (Ben-Shahar et al., 2000; Komischke et al., 2002; Hadar and Menzel, 2010; Mota and Giurfa, 2010). Pharmacological blocking of the mushroom bodies (MBs), higher-order brain structures associated with memory storage and retrieval (Menzel, 1999, 2014; Giurfa and Sandoz, 2012) impairs reversal learning but leaves intact the capacity to achieve two successive elemental olfactory discriminations (Devaud et al., 2007).

Here, we aimed at uncovering the mechanisms underlying the necessity of MBs for reversal learning. We focused on the Gamma-AminoButyric Acid (GABA)ergic signals provided by A3v and A3d neurons, which provide the only inhibitory feedback circuits known so far in the MBs of the honey bee (Bicker et al., 1985; Gronenberg, 1987; Grünewald, 1999a,b). Both innervate the output region of the MBs (the lobes); A3v neurons feedback onto the input region (the calyces) of the MBs and A3d neurons feedback onto the lobes themselves. We reasoned that these circuits might be crucial to inhibit responses to previously reinforced odors during the reversal phase. We thus blocked GABAergic signaling in the MBs during olfactory reversal learning by locally injecting antagonists of ionotropic or metabotropic GABA receptors, into the calyces or the lobes. In this way, we aimed at determining the GABA receptors relevant for this task. We show that only ionotropic GABAergic signaling in the calyces is required for reversal learning but not for two consecutive elemental discriminations. We thus provide a circuit-based explanation of the implication of MBs in reversal learning and discuss the specialization of distinct brain areas in learning forms of variable complexity.

## Materials and Methods

### Animal Preparation

Female honey bee foragers (*Apis mellifera*) were captured at the entrance of the hive in the morning of each experimental day. To be handled properly, they were anaesthetized on ice for a few minutes until complete immobilization. They were then harnessed individually in small metal tubes. Only the antennae and the mouthparts remained free to move (Bitterman et al., 1983). This preparation ensures optimal conditions for the olfactory conditioning of the proboscis extension reflex (PER; Bitterman et al., 1983; Matsumoto et al., 2012) which was used in our experiments. In this protocol, harnessed bees, which extend the proboscis upon antennal stimulation with sucrose solution (unconditioned stimulus or US), learn to associate a neutral odorant (conditioned stimulus or CS) with the sucrose delivered to the antennae and the proboscis, and respond afterwards with a PER to the conditioned odorant (Bitterman et al., 1983; Giurfa and Sandoz, 2012; Matsumoto et al., 2012). As PER conditioning was combined with localized microinjections of GABAergic antagonists in the bee brain, a piece of cuticle was removed from the head to expose the brain and allow later injections. The brain was then accessible through a window located between the compound eyes, the antennae and the median ocellus. The piece of cuticle was then put back in its original position to avoid brain desiccation. Bees were fed with 5 μL of sucrose solution (50% weight/weight) before being stored in a dark humid chamber at room temperature for 3 h in order to allow recovery until the experiment.

### Injections

Picrotoxin (PTX 5 μM, Sigma-Aldrich France) and CGP_54626_ (250 μM, RnD Systems France) were used to block ionotropic GABA (Froese et al., 2014) and metabotropic GABA (Dupuis et al., 2010) receptors, respectively. We used the procedure established by Devaud et al. (2007) for microinjections in the MBs of the honey bee. Briefly, drugs were dissolved in a phosphate buffer saline solution (PBS) which was also used as a control. The PBS (in mM) solution consisted of: sucrose, 160; glucose, 25; HEPES, 10; MgCl_2_, 4; NaCl, 130; KCl, 6; CaCl_2_, 5 (pH 6.7). Methylene blue (1 mM, Sigma-Aldrich France) was added to the solutions to visualize and control the success and correct location of injections. The volume of the injected solution was first calibrated by injecting the solution into a drop of mineral oil using a Malassez cell. Depending on the experiment, a volume of 0.5 nL of either PTX, CGP_54626_ or PBS was injected bilaterally, between the lateral and median calyces, or in the vertical lobes of the MBs using a pulled glass capillary (GC 100–10, Harvard Apparatus, Les Ulis, France) connected to a pressure micro-injector (FemtoJet express, Eppendorf, France). The injection was performed 45 min after the first phase and 15 min before the second phase.

### Preliminary Experiments

**Figure 1:**
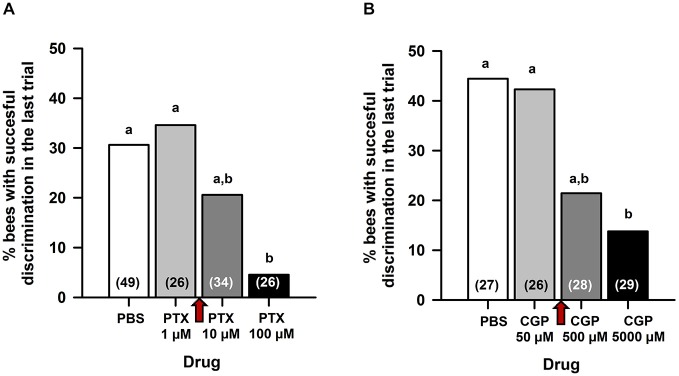
**Preliminary experiments for choosing the concentration of picrotoxin (PTX) and CGP_54626_ to be used in the reversal learning experiments.** Fifteen minutes before conditioning, bees were injected in the MB calyces with either PBS or one of the two antagonists, and subject to a classical differential conditioning with a rewarded odor A and an unrewarded odor B (A^+^ vs. B^−^). Sample sizes are indicated in parentheses within bars. **(A)** In the case of PTX, three concentrations were assayed: 1, 10 and 100 μM. The percentage of bees exhibiting successful discrimination (i.e. responding to odor A and not to odor B in the last conditioning trial) varied with the concentration of PTX tested. Different capital letters above bars indicate significant differences. PBS-injected bees and bees injected with PTX 1 μM learned the discrimination and reached comparable levels of discrimination (test for comparison of multiple proportions; NS). Bees injected with PTX 100 μM did not learn the discrimination and differed significantly from PBS-injected bees and from bees injected with PTX 1 μM. The concentration of PTX 10 μM yielded intermediate results. A concentration of PTX 5 μM (red arrow between 1 and 10 μM) was thus chosen for our experiments. **(B)** In the case of CGP_54626_, three concentrations were assayed: 50, 500 and 5000 μM. The % of bees exhibiting successful discrimination (i.e. responding to odor A and not to odor B in the last conditioning trial) varied with the concentration of CGP_54626_ tested. Different capital letters above bars indicate significant differences. PBS-injected bees and bees injected with CGP_54626_ 50 μM learned the discrimination and reached comparable levels of discrimination (test for comparison of multiple proportions; NS). Bees injected with CGP_54626_ 5000 μM showed deficient discrimination learning and differed significantly from PBS-injected bees. The concentration of CGP_54626_ 500 μM yielded intermediate results. Thus, a concentration of 250 μM CGP5_4626_ (red arrow between 50 and 500 μM) was chosen for our experiments.

As MB blockade by procaine impairs reversal but not differential conditioning (Devaud et al., 2007), we reasoned that a similar effect could be obtained after PTX and/or CGP_54626_ injection if the GABAergic signaling targeted by these drugs underlies the procaine effect. Pilot experiments (Figure 1) were therefore designed to define the concentrations of PTX and CGP_54626_ necessary to target efficiently ionotropic and metabotropic GABA receptors, respectively, while leaving intact the capacity to learn a simple olfactory discrimination. Three hours after opening a window in the head capsule (see above) and 15 min before conditioning, bees were injected in the MB calyces with either PBS or one of the two antagonists, and subject to a classical differential conditioning with a rewarded odor A and an unrewarded odor B (A^+^ vs. B^−^). In the case of PTX (Figure 1A), which blocks ionotropic GABA receptors, three concentrations were assayed, each in an independent group of bees: 1 μM, 10 μM or 100 μM. The higher PTX concentration was chosen as it was shown to abolish inhibitory GABAergic signaling when injected into the antennal lobes (ALs) of honey bees (Stopfer et al., 1997), locusts (MacLeod and Laurent, 1996), moths (Mwilaria et al., 2008) and fruit flies (Wilson and Laurent, 2005) before a differential conditioning. In all cases, control bees injected with PBS learned the olfactory discrimination. PTX-injected bees also learned the discrimination at a concentration of 1 μM but not at 100 μM. The concentration of 10 μM yielded intermediate results. We thus chose the concentration of 5 μM PTX (between 1 and 10 μM) for our experiments. In the case of CGP_54626_ (Figure 1B), which blocks metabotropic GABA receptors, three concentrations were assayed, each in an independent group of bees: 50, 500 or 5000 μM. The concentration of CGP_54626_ 500 μM is known to affect the response of bathed Kenyon cells (KCs) *in vivo* upon odorant delivery (Froese et al., 2014). In all cases, control bees injected with PBS learned the olfactory discrimination. CGP_54626_-injected bees learned the discrimination at a concentration of 50 μM but not at 5000 μM; bees injected with the concentration of 500 μM had an intermediate discrimination. Thus, a concentration of 250 μM CGP_54626_ (between 50 and 500 μM) was chosen for our experiments.

### Experiments

**Figure 2:**
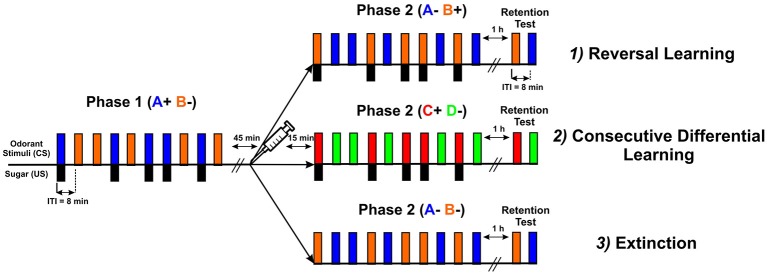
**Scheme describing the three types of experiments performed: (1) reversal learning (A^+^ vs. B^−^ → A^−^ vs. B^+^); (2) consecutive differential learning (A^+^ vs. B^−^ → C^+^ vs. D^−^); and (3) extinction (A^+^ vs. B^−^ → A^−^ and B^−^).** All three types had the same common first phase, in which bees were trained to discriminate two odorants (CS), one (blue bars) paired five times with sucrose delivery (black bars, US) and the other presented five times without sucrose (orange bars). The intertrial interval (ITI) was 8 min. After the first phase, bees experienced a 45 min rest followed by an injection of PBS, PTX or CGP_54626_. The second conditioning phase started 15 min after injection. In the reversal learning experiment, the same odorants were presented with a reversed contingency. In the consecutive differential-learning experiment, odorants A and B were replaced by two novel odorants C and D. In the extinction experiment, odorants A and B were presented again but without sucrose reward. In all three protocols, a retention test was performed 1 h after the end of the second phase. In this test, the two odorants used in the last conditioning phase were presented without sucrose reinforcement.

Three types of experiments were performed (Figure 2): (1) *reversal learning* (A^+^ vs. B^−^ → A^−^ vs. B^+^); (2) *consecutive differential learning* (A^+^ vs. B^−^ → C^+^ vs. D^−^); and (3) *extinction* (A^+^ vs. B^−^ → A^−^ and B^−^). In all three experiments, each conditioning phase consisted of five rewarded and five non-rewarded trials presented in a pseudorandomized sequence (10 trials in total), except in the extinction protocol where all trials were unrewarded (five with each odorant) in the second phase. The intertrial interval (ITI) was 8 min. In all three cases, only bees that learned the first discrimination (i.e., that responded more to the rewarded than to the non-rewarded odorant) were used (Roussel et al., 2010; Pamir et al., 2011), as studying reversal learning is only meaningful in the case of first-phase effective learners (Mota and Giurfa, 2010; de Brito Sanchez et al., 2015). The first phase of all three experiments was identical and consisted of an olfactory differential conditioning with two odors, limonene ([R]-(+)-limonene 97%, Sigma-Aldrich France) and eugenol (eugenol ≥ 99%, Sigma-Aldrich France), one rewarded with a 50% (weight/weight) sucrose solution (A^+^) and the other non-rewarded (B^−^). Odorants were chosen to reproduce the conditions of Devaud et al. (2007) and their roles as A or B were balanced. After the first phase, bees experienced a 45 min rest followed by an injection of PBS, PTX or CGP_54626_. The second conditioning phase started 15 min after injection. In the reversal learning experiment, the same odors were presented with a reversed contingency, as odor A was no longer rewarded while odor B was rewarded (phase 2: A^−^ vs. B^+^) thus creating a transient ambiguity of stimulus valence. In the experiment using two consecutive differential learning phases, A and B were replaced by two novel odors C (1-heptanal ≥ 99%, Sigma-Aldrich France) and D (1-nonanol ≥ 99%, Sigma-Aldrich France) in the second phase (phase 2: C^+^ vs. D^−^). In the extinction experiment, odors A and B were also presented in the second phase but without reward (phase 2: A^−^ and B^−^). In all three protocols, a retention test was performed 1 h after the end of the second phase. In this test, the two odorants used in the last conditioning phase were presented without sucrose reinforcement. The inter-test interval was also 8 min. The sequence of odorant presentations was randomized from bee to bee.

### Conditioning Procedure

We followed the standard procedures described in Matsumoto et al. (2012). In all experiments, only bees responding with a PER to the sucrose stimulus before conditioning and after the retention test were used. Each conditioning trial lasted 40 s, starting when the bee was positioned in front of the odor-delivery system sending a clean air flow at 90–100 mL/min. Fifteen seconds after the start of the trial, the odorant was delivered by a computer-controlled olfactometer during 4 s by diverting the air flow through a syringe containing a filter paper soaked with 4 μL of pure odorant. To induce a PER in the case of the rewarded odor presentations, both antennae were simultaneously touched with a toothpick soaked with 50% sucrose solution during 3 s, starting 3 s after the odor onset. Therefore, the inter-stimulus interval was 3 s and the stimulus overlap was 1 s.

### Statistical Analysis

All results are presented as percentages of bees exhibiting a PER to the conditioned odorants. The data met the conditions required to apply an ANOVA to a dichotomous dependent variable (Lunney, 1970; D’Agostino, 1971) and thus allowed the use of repeated-measurement ANOVA for comparisons between and within groups. A Tukey honest significant difference (HSD) *post hoc* analysis was performed after ANOVA to specify statistical differences. The statistics reported for the first phase of each experiment refer to the selected group of bees which learned the discrimination and which entered the second phase. All statistical analyses were performed using Statistica 7.1 (StatSoft France).

## Results

### Experiment 1: Blocking Ionotropic, but not Metabotropic, GABAergic Signaling into the MB Calyces Impairs Reversal Learning

**Figure 3:**
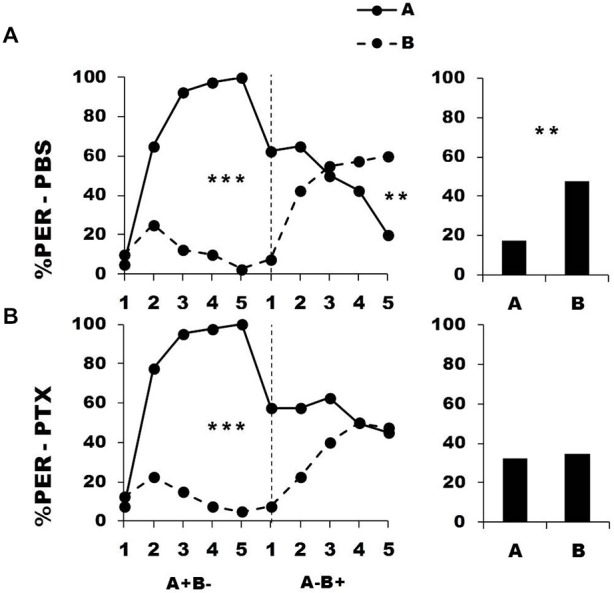
**Blockade of ionotropic Gamma-AminoButyric Acid (GABA) receptors in the calyces of the mushroom bodies impairs reversal learning.** Percentage of PER induced by odor A (*solid line*) and odor B (*dotted line*) during the first (A^+^ vs. B^−^) and the second phase (A^−^ vs. B^+^) of a reversal conditioning, after injections of either saline [PBS, *n* = 40, **(A)**] or picrotoxin solution [PTX, *n* = 40, **(B)**], which blocks ionotropic GABA receptors. A retention test without reinforcement was performed 1 h after the end of the second phase. **p < 0.01; ***p < 0.001 (Tukey honest significant difference, HSD *post hoc* analysis on the last trial of each phase and retention test).

Bees were trained during a first conditioning phase to discriminate a rewarded odorant A from a non-rewarded odorant B (A^+^ vs. B^−^). Afterwards, they were injected with either PBS (control group) or PTX, which blocks ionotropic GABA receptors, and subjected to a second conditioning phase in which odorant contingencies were reversed (A^−^ vs. B^+^). Figure 3 shows the performance of bees that effectively learned the olfactory discrimination of the first phase (85% of trained individuals). In the first conditioning phase, and prior to injection, both the PBS (*n* = 40; Figure 3, upper panel) and the PTX group (*n* = 40, Figure 3, lower panel) learned the discrimination between A^+^ and B^−^ (PBS, factor odorant: *F*_1,39_ = 229.41, *p* = 0.0001; PTX, factor odorant: *F*_1,39_ = 248.26, *p* = 0.0001; repeated-measure ANOVA). In both groups, differentiation between A^+^ and B^−^ occurred from the second trial onwards (Tukey HSD *post hoc* analysis: *p* = 0.0001). The interaction trial *x* odorant was also significant (PBS: *F*_4,39_ = 49.73, *p* = 0.0001; PTX: *F*_4,39_ = 49.34, *p* = 0.0001) thus showing that responses to A^+^ and B^−^ followed different trends in both groups. Importantly, a comparison between the PBS and the PTX groups at this stage showed no significant difference in their acquisition performances (factor group: *F*_2,77_ = 0.69, *p* = 0.51) thus confirming that the learning of the first-phase discrimination was identical between groups prior to drug injection.

After injection, PBS-injected bees successfully learned the discrimination of the second phase (trial *x* odorant interaction: *F*_4,39_ = 29.26, *p* = 0.0001) and thus achieved the reversal learning by responding more to B^+^ than to A^−^ in the last trial (Tukey HSD for the last trial: *p* = 0.006). One hour after the end of the second conditioning phase, PBS-injected bees retained the last information learned and responded significantly more to B than to A (Figure 3, upper bar diagram: *F*_1,39_ = 9.85, *p* = 0.003). On the contrary, PTX-injected bees exhibited impaired reversal learning (trial *x* odorant interaction: *F*_4,39_ = 9.33, *p* = 0.0001) as they did not respond differently to both odorants in the last trial (Tukey HSD: *p* = 0.99). In the retention test performed 1 h after the end of the second phase, these bees were still unable to differentiate between odorants A and B (Figure 3, lower bar diagram: *F*_1,39_ = 0.11, *p* = 0.74), consistently with their incapacity to fully reverse odorant contingencies. Thus, inhibition of ionotropic GABA receptors at the level of the calyces *via* PTX impaired the capacity to achieve olfactory reversal learning in honey bees.

An important question in the case of the PTX-injected bees is whether olfactory reversal was indeed impaired by PTX or was simply delayed as could suggest the apparent decrease of responses to odorant A in the second conditioning phase. Contrarily to PBS-injected bees, the responses to A of the PTX group did not decrease during this phase (PBS group: *F*_4,39_ = 12.90, *p* < 0.001; PTX group: *F*_4,39_ = 2.02, *p* = 0.55). This shows that the decrease of responses to A was only apparent and different from that observed in the PBS-injected bees. The responses of PTX-injected bees in the retention test confirmed this conclusion: this test reflects the cumulative experience with A and B gathered up to trial 5^th^ and allows further measuring of conditioned responses to A and B. Moreover, the time elapsed (1 h) between the end of the second phase and the retention test should favor further consolidation and reversal. However, the test performance of the PTX group did not show any sign of successful reversal. Response levels of the PTX group in the last trial of the second conditioning phase and in the retention test remained identical both for odorant A (PTX: *F*_1,39_ = 1.69, *p* = 0.20) and B (PTX: *F*_1,39_ = 1.97, *p* = 0.17). It is thus possible to conclude that the performance of the PTX-injected bees was not consistent with a delayed reversal but reflected a real impairment of the reversal process.

**Figure 4:**
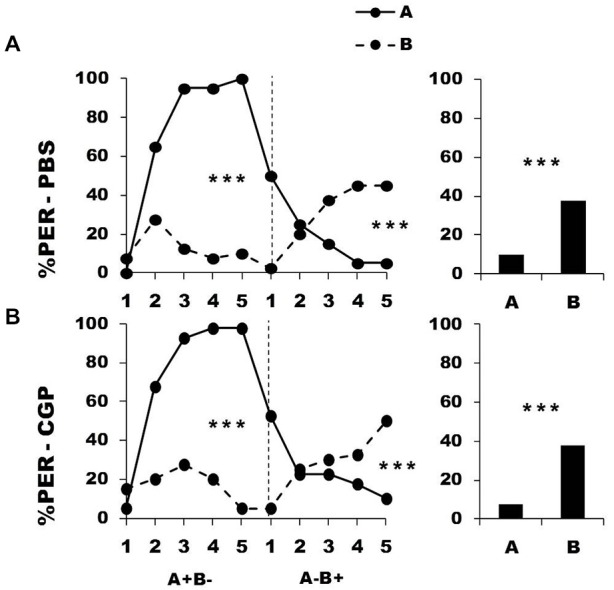
**Blockade of metabotropic GABA receptors in the calyces of the mushroom bodies does not affect reversal learning.** Percentage of PER induced by odor A (*solid line*) and odor B (*dotted line*) during the first (A^+^ vs. B^−^) and the second phase (A^−^ vs. B^+^) of a reversal conditioning, after injections of either saline [PBS, *n* = 40, **(A)**] or CGP_54626_ [CGP, *n* = 40, **(B)**], which blocks metabotropic GABA receptors. ***p < 0.001 (Tukey HSD *post hoc* analysis on the last trial of each phase and retention test).

In a further experiment, we blocked metabotropic GABA receptors in the calyces *via* CGP_54626_ injection performed between the two conditioning phases. Figure 4 shows the performance of bees that effectively learned the olfactory discrimination of the first phase (87% of trained individuals). In the first conditioning phase, and prior to injection, both the PBS group (*n* = 40; Figure 4, upper panel) and the CGP_54626_ group (*n* = 40; Figure 4, lower panel) learned the discrimination between A^+^ and B^−^ (PBS: factor odorant: *F*_1,39_ = 222.37, *p* = 0.0001; CGP_54626_: *F*_1,39_ = 141.31, *p* = 0.0001). In both groups, differentiation between A^+^ and B^−^ occurred from the second trial onwards (Tukey HSD: *p* = 0.0001) and the interaction trial *x* odorant was significant (PBS group: *F*_4,39_ = 49.73, *p* = 0.0001; CGP_54626_: *F*_4,39_ = 36.66, *p* = 0.0001). Again, the two groups showed no difference in acquisition during this first phase (factor group: *F*_2,77_ = 0.61, *p* = 0.55) thus confirming that their performances were identical prior to drug injection. After injection, both PBS and CGP_54626_-injected bees successfully learned the discrimination of the second phase (trial *x* odorant interaction, PBS: *F*_4,39_ = 30.85, *p* = 0.0001; CGP_54626_: *F*_4,39_ = 21.21, *p* = 0.0001) and responded significantly more to B^+^ than to A^−^ in the last trial (Tukey HSD; PBS: *p* = 0.0004; CGP_54626_: *p* = 0.0009). One hour after the end of the second conditioning phase, both groups of bees retained the last information learned and responded significantly more to the odorant B than to A (PBS: *F*_1,39_ = 14.79, *p* = 0.0004; CGP_54626_: *F*_1,39_ = 16.71, *p* = 0.0002). Furthermore, the curves of responses to A and B in the second conditioning phase did not differ between the PBS group and the CGP_54626_ group (odorant A: *F*_1,39_ = 0.57, *p* = 0.45; odorant B: *F*_1,39_ = 0.04, *p* = 0.84). Thus, inhibition of metabotropic GABA receptors at the level of the calyces *via* CGP_54626_ did not affect the capacity to achieve olfactory reversal learning in honey bees.

Overall, these results suggest that successful olfactory reversal learning in honey bees requires GABAergic signaling through ionotropic but not metabotropic GABA receptors located in the calyces of the MBs.

### Experiment 2: Blocking Ionotropic GABAergic Signaling into the MB Calyces does not Impair the Acquisition of Two Consecutive Differential Discriminations

We next determined if the impairment of reversal learning induced by PTX was specific to this task, and thus to the ambiguity of stimulus valence created by the reversal of stimulus contingencies in the second conditioning phase, or could also occur in the case of two consecutive elemental discriminations with no ambiguity of stimulus valence. To this end, bees were trained during a first conditioning phase to discriminate a rewarded odorant A from a non-rewarded odorant B (A^+^ vs. B^−^), afterwards they were injected into the MB calyces with either PBS or PTX, and then they were subjected to a second conditioning phase with two different odorants C and D, one rewarded and the other non-rewarded (C^+^ vs. D^−^). Finally, the retention test was performed as previously. CGP_54626_ was not used in this experiment given its failure to block reversal learning (see “Experiment 1: Blocking ionotropic, but not metabotropic, GABAergic signaling into the MB calyces impairs reversal learning” Section).

**Figure 5:**
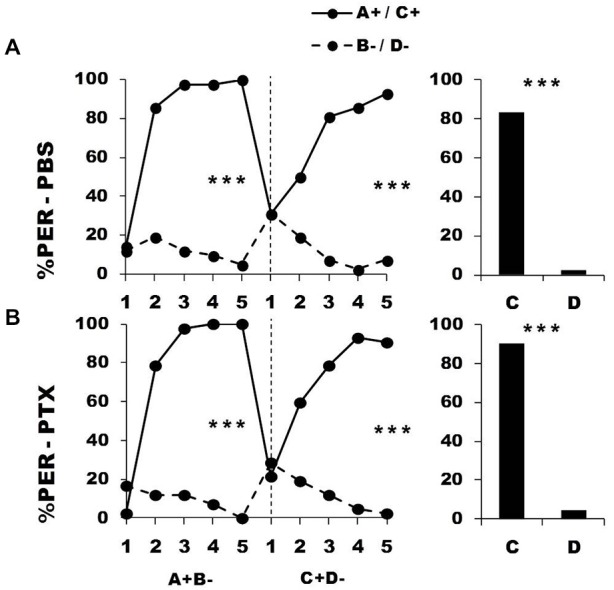
**Blockade of ionotropic GABA receptors in the calyces of the mushroom bodies does not affect learning of two consecutive differential discriminations.** Percentage of PER induced by odors A and C (*solid lines*) and odors B and D (*dotted lines*) during the first (A^+^ vs. B^−^) and the second conditioning phase (C^+^ vs. D^−^), after injections of either saline [PBS, *n* = 42, **(A)**] or picrotoxin solution [PTX, *n* = 42, **(B)**], which blocks ionotropic GABA receptors. ***p < 0.001 (Tukey HSD *post hoc* analysis on the last trial of each phase and retention test).

Figure 5 shows the performance of bees that effectively learned the olfactory discrimination of the first phase (87% of trained individuals). In the first conditioning phase, and prior to injection, both the PBS (*n* = 42; Figure 5, upper panel) and the PTX group (*n* = 42; Figure 5, lower panel) learned to discriminate A^+^ from B^−^ (repeated-measure ANOVA, factor odorant, PBS: *F*_1,41_ = 265.31, *p* = 0.0001; PTX: *F*_1,41_ = 502.32, *p* = 0.0001). In both groups, differentiation between A^+^ and B^−^ occurred from the second trial onwards (Tukey HSD: *p* = 0.0001) and the interaction trial *x* odorant was significant (PBS: *F*_4,41_ = 54.53, *p* = 0.0001; PTX: *F*_4,41_ = 79.19, *p* = 0.0001), thus showing that responses to A^+^ and B^−^ followed different trends in both groups. As in the previous experiment, the PBS and the PTX groups did not differ at this stage (*F*_2,81_ = 1.09, *p* = 0.34), thus confirming that the first discrimination learning was identical between groups prior to drug injection. After injection, both groups successfully learned the discrimination C^+^ D^−^ of the second phase (trial *x* odorant interaction, PBS: *F*_4,41_ = 33.64, *p* = 0.0001; PTX: *F*_4,41_ = 38.43, *p* = 0.0001) in a similar way, and responded significantly more to C^+^ than to D^−^ in the last trial (Tukey HSD: *p* = 0.0001 in both cases). One hour after the end of the second conditioning phase, both groups of bees retained the last information learned and responded significantly more to the odorant C than to D (PBS: *F*_1,41_ = 174.25, *p* = 0.0001; PTX: *F*_1,41_ = 246.00, *p* = 0.0001). Thus, despite its significant effect on reversal learning, GABAergic inhibition by PTX injection at the level of the calyces did not affect the ability to learn two consecutive elemental discriminations with no ambiguity of stimulus valence.

### Experiment 3: Impairment of Olfactory Reversal Learning Through PTX Injection into the Calyces is Neither Due to Impaired Retrieval nor to Impaired Extinction

Two possible indirect effects could explain the impairment of reversal learning in PTX-injected bees as observed in Figure 3. First, the injection of PTX could have impaired retrieval and thus resulted in a reduction of the initial level of responses to A between the end of the first phase and the first trial of the second phase. In this case, a further decrease resulting from reversal might no longer be observed along the second phase. This possibility can be discarded as the decrease in responses to the odorant A in the first trial of the second phase, as compared to the last trial of the first phase, was similar in both groups (PTX: 58% PER, PBS: 63% PER; *F*_1,39_ = 0.05, *p* = 0.82), and nevertheless allowed successful reversal in the PBS group. Thus, PTX did not impair specifically odorant retrieval. Second, the injection of PTX might have impaired extinction learning. Indeed, reversal learning requires that the response to the formerly non-rewarded stimulus B increases while the response to the previously rewarded stimulus A extinguishes.

As PTX-injected bees did not diminish their responses to A when no longer rewarded, the impairment might be explained by a failure of extinction learning of A when no longer rewarded (Figure 3: 2nd phase). We thus tested whether extinction was impaired in these animals by repeatedly presenting odorants A and B without sucrose during the second conditioning phase (A^−^ and B^−^).

**Figure 6:**
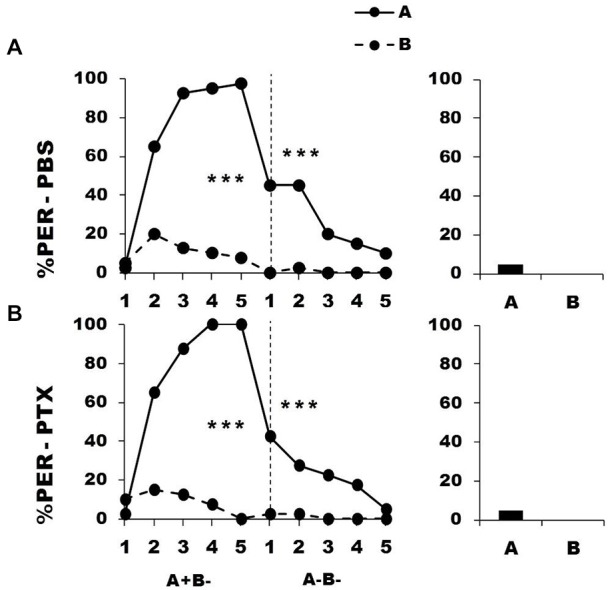
**Blockade of ionotropic GABA receptors in the calyces of the mushroom bodies does not affect extinction learning.** Percentage of proboscis extension response (PER) induced by odor A (*solid line*) and odor B (*dotted line*) during the first conditioning phase (A^+^ vs. B^−^) and the second extinction phase (A^−^ and B^−^), after injections of either saline [PBS, *n* = 40, **(A)**] or picrotoxin solution [PTX, *n* = 40, **(B)**], which blocks ionotropic GABA receptors. The levels of significance shown for the extinction phase correspond to the decrease of responses to odor A along trials; repeated-measure ANOVA. ***p < 0.001 (Tukey HSD *post hoc* analysis on the last trial of each phase and retention test).

Figure 6 shows the performance of bees that effectively learned the olfactory discrimination A^+^ vs. B^−^ of the first phase (89% of trained individuals). As expected, in this first phase, and prior to injection, both the PBS (*n* = 40; Figure 6, upper panel) and the PTX group (*n* = 40; Figure 6, lower panel) learned to discriminate A^+^ from B^−^ (repeated-measure ANOVA, factor odorant, PBS: *F*_1,39_ = 276.83, *p* = 0.0001; PTX: *F*_1,39_ = 418.76, *p* = 0.0001). In both groups, differentiation between A^+^ and B^−^ occurred from the second trial onwards (Tukey HSD: *p* = 0.0001) and the interaction trial *x* odorant was significant (PBS: *F*_4,39_ = 38.09, *p* = 0.0001; PTX: *F*_4,39_ = 50.68, *p* = 0.0001). Again, the PBS and the PTX groups did not differ at this stage (factor group: *F*_2,77_ = 0.39, *p* = 0.68), thus confirming that the first discrimination learning was identical between groups prior to drug injection.

After injection, and during the second phase (extinction phase, A^−^ B^−^), both groups showed a significant decrease in their response to the non-rewarded odorant A (PBS: *F*_4,39_ = 9.64, *p* = 0.0001; PTX: *F*_4,39_ = 6.48, *p* = 0.0001). In both cases, extinction was almost complete at the end of the second phase, as the responses in the 5th trial did not differ from 0 (*t*-test: *t*_1, 39_ = 1.43, *p* = 0.16). The decrease of the responses to A was statistically equivalent in the two groups (group effect: *F*_1,39_ = 0.51, *p* = 0.48; group *x* trial interaction: *F*_4,39_ = 1.46, *p* = 0.21), thus showing that extinction was preserved in the PTX-injected bees and was comparable to that of PBS-injected bees. As expected, responses to B^−^ remained at the low level already reached at the end of the first phase and did not vary along the second phase (*F*_4,39_ = 1.00, *p* = 0.41 for both groups). Due to this, bees of both groups did not respond differently to A and B in the retention test performed 1 h after the end of conditioning (*F*_1,39_ = 2.05, *p* = 0.16 for both groups).

Taken together, these results indicate that olfactory memory retrieval (accessible through the first trial of the second phase) as well as extinction learning (accessible through the variation of responses to A in the second phase) were unaffected by PTX injection into the calyces of the MBs. Therefore, impairment of reversal learning was not due to the impossibility to learn changes in reinforcement outcome. Rather, PTX blockade seems to affect specifically the processes necessary to achieve *reversal* of stimulus valence.

### Experiment 4: Blocking Ionotropic GABAergic Signaling into the MB Lobes Impairs Neither Reversal Learning nor Two Consecutive Differential Discriminations

In the honey bee brain, two GABAergic subpopulations of neurons provide inhibitory feedback to the MBs (Bicker et al., 1985; Rybak and Menzel, 1993; Grünewald, 1999a; Ganeshina and Menzel, 2001): A3v neurons arborize in the vertical lobe and project to the calyces, mainly to the basal ring and the lip (Bicker et al., 1985; Rybak and Menzel, 1993), while A3d neurons arborize mainly in the medial lobe and project to the vertical lobe (Bicker et al., 1985). While our previous experiments targeted GABAergic signaling into the calyces, and thus most probably A3v neuron signaling, it remained to determine whether the potential GABAergic contribution of A3d neurons was equally important for reversal learning. To study the possible implication of these neurons (i.e., of GABAergic signaling into the vertical lobes) in olfactory reversal learning, we coupled reversal conditioning with an injection of either PBS or PTX into the MB vertical lobes. The injection was performed as in the previous experiments, between the two conditioning phases.

**Figure 7:**
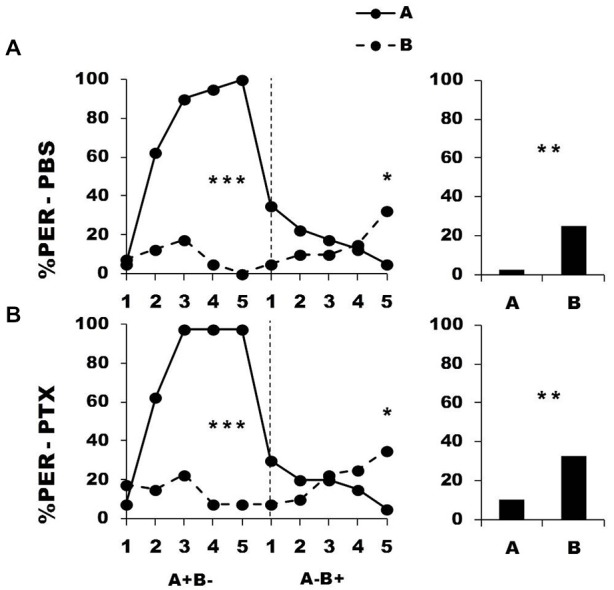
**Blockade of ionotropic GABA receptors in the lobes of the mushroom bodies does not impair reversal learning.** Percentage of PER induced by odor A (*solid line*) and odor B (*dotted line*) during the first (A^+^ vs. B^−^) and the second phase (A^−^ vs. B^+^) of reversal conditioning, after injection of either saline [PBS, *n* = 40, **(A)**] or picrotoxin solution [PTX, *n* = 40, **(B)**], which blocks ionotropic GABA receptors. *p < 0.05; **p < 0.01; ***p < 0.001 (Tukey HSD *post hoc* analysis on the last trial of each phase and retention test).

Figure 7 shows the performance of bees that effectively learned the olfactory discrimination A^+^ vs. B^−^ of the first phase (86% of trained individuals). In this first phase, and prior to injection, both the PBS (*n* = 40; Figure 7, upper panel) and the PTX group (*n* = 40; Figure 7, lower panel) learned to discriminate A^+^ from B^−^ (repeated-measure ANOVA, factor odorant, PBS: *F*_1,39_ = 146.75, *p* = 0.0001; PTX: *F*_1,39_ = 180.91, *p* = 0.0001). In both groups, differentiation between A^+^ and B^−^ occurred from the second trial onwards (Tukey HSD: *p* = 0.0001) and the interaction trial *x* odorant was significant (PBS: *F*_4,39_ = 38.63, *p* = 0.0001; PTX: *F*_4,39_ = 46.49, *p* = 0.0001), thus showing that responses to A^+^ and B^−^ followed different trends in both groups. As in the previous experiment, the PBS and the PTX groups did not differ at this stage (*F*_2,77_ = 0.82, *p* = 0.44), thus confirming that the first discrimination learning was identical between groups prior to drug injection. After injection, both the PBS and the PTX groups successfully learned the discrimination A^−^ vs. B^+^ of the second phase (trial *x* odorant interaction, PBS: *F*_4,39_ = 12.64, *p* = 0.0001; PTX: *F*_4,39_ = 12.55, *p* = 0.0001) and responded significantly more to B^+^ than to A^−^ in the last trial (Tukey HSD: *p* = 0.02 in both cases). Both groups exhibited similar learning patterns during the second phase (repeated-measure ANOVA, factor group: *F*_2,77_ = 0.17, *p* = 0.84). One hour after the end of the second conditioning phase, both groups of bees retained the last information learned and responded significantly more to the odorant A than to B (*F*_1,39_ = 11.32, *p* = 0.002 for both groups).

Thus, inhibition of GABAergic signaling into the vertical lobe did not affect the ability to master reversal learning. In other words, since ionotropic GABA receptors are necessary to achieve this task in the calyces but not in the lobes, this suggests that A3d neurons are dispensable for reversal learning, contrary to A3v neurons. If this conclusion is valid, then ionotropic GABAergic feedback to the lobes *via* A3d neurons may also be dispensable for successive conditioning of two simple olfactory discriminations. To test this hypothesis, we coupled the successive conditioning of two simple discriminations (A^+^ B^−^ → C^+^ D^−^) with an injection of either PBS or PTX into the MB vertical lobes. The injection was performed as before, between the two conditioning phases.

**Figure 8:**
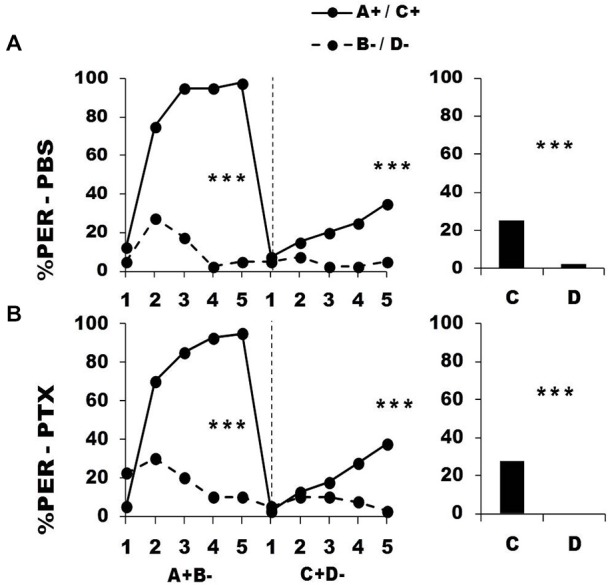
**Blockade of ionotropic GABA receptors in the lobes of the mushroom bodies does not affect learning of two consecutive differential discriminations.** Percentage of PER induced by odors A and C (*solid lines*) and odors B and D (*dotted lines*) during the first (A^+^ vs. B^−^) and the second conditioning phase (C^+^ vs. D^−^), after injections of either saline [PBS, *n* = 42, **(A)**] or picrotoxin solution [PTX, *n* = 42, **(B)**], which blocks ionotropic GABA receptors. ***p < 0.001 (Tukey HSD *post hoc* analysis on the last trial of each phase and retention test).

Figure 8 shows the performance of bees that effectively learned the olfactory discrimination A^+^ vs. B^−^ of the first phase (92% of trained individuals). In this first phase, and prior to injection, both the PBS (*n* = 40; Figure 8, upper panel) and the PTX group (*n* = 40; Figure 8, lower panel) learned to discriminate A^+^ from B^−^ (repeated-measure ANOVA, factor odorant, PBS: *F*_1,39_ = 227.01, *p* = 0.0001; PTX: *F*_1,39_ = 89.49, *p* = 0.0001). In both groups, differentiation between A^+^ and B^−^ occurred from the second trial onwards (Tukey HSD: *p* = 0.0001) and the interaction trial *x* odorant was significant (PBS: *F*_4,39_ = 38.56, *p* = 0.0001; PTX: *F*_4,39_ = 33.58, *p* = 0.0001), thus showing that responses to A^+^ and B^−^ followed different trends in both groups. As in the previous experiment, the PBS and the PTX groups did not differ at this stage (factor group: *F*_2,77_ = 1.06, *p* = 0.35), thus confirming that the first discrimination learning was identical between groups prior to drug injection.

After injection, both the PBS and the PTX groups successfully learned the discrimination C^+^ D^−^ of the second phase (trial *x* odorant interaction, PBS: *F*_4,39_ = 3.05, *p* = 0.002; PTX: *F*_4,39_ = 5.05, *p* = 0.0005) and responded significantly more to C^+^ than to D^−^ in the last trial (Tukey HSD: *p* = 0.0001 in both cases). Both groups exhibited similar learning patterns during the second phase (repeated-measure ANOVA, factor group: *F*_2,77_ = 0.04, *p* = 0.96). One hour after the end of the second conditioning phase, both groups of bees retained the last information learned and responded significantly more to the odorant C than to D (PBS: *F*_1,39_ = 11.32, *p* = 0.0001; PTX: *F*_1,39_ = 13.21, *p* = 0.0001).

The last two experiments exhibit lower response rates during the second conditioning phase when compared to the corresponding experiments in which PBS and PTX were injected into the calyces (compare Figures 3, 7 for reversal learning, and Figures 5, 8 for consecutive differential conditioning). This effect was common to the PBS and the PTX groups, which in all cases showed similar performances in the second conditioning phase (see Figures 7, 8) and could be due to a damage done to the lobes by the injection needle, leading to a possible impairment of retrieval processes localized in part in the lobes (Lozano et al., 2001) and/or of signaling from the lobes to the premotor areas (e.g., lateral horn) which control PER.

## Discussion

We studied the involvement of the MBs, higher-order structures of the insect brain, in reversal learning, a form of learning that requires overcoming the temporary ambiguity of stimulus valence that underlies the transition between two conditioning phases with opposed stimulus valences. MBs were previously shown to be necessary for solving this task in honey bees as pharmacological blockade of voltage-gated Na^+^ and K^+^ currents by means of local injections of the anesthetic procaine impaired reversal learning but not the capacity to solve two consecutive simple olfactory discriminations with no ambiguity of stimulus valence (Devaud et al., 2007). Here we confirmed the necessity of MBs for reversal learning and further uncovered the mechanism and potential circuits underlying this effect. We show that inhibition of ionotropic but not metabotropic GABAergic signaling into the MB calyces impairs reversal learning, yet leaves intact the capacity to perform two consecutive elemental olfactory discriminations without ambiguity of stimulus valence. On the contrary, inhibition of ionotropic GABAergic signaling into the MB lobes had no effect on reversal learning. Our results thus suggest that A3v neurons providing ionotropic GABAergic feedback to the MB calyces, but not to the lobes themselves, seem to be specifically required for counteracting ambiguity of stimulus valence in reversal learning.

The importance of GABAergic signaling has been demonstrated in studies of appetitive and aversive conditioning in bees (Raccuglia and Mueller, 2013) and fruit flies (Liu et al., 2007; Liu and Davis, 2009), respectively. Using simple associative conditioning protocols, it was shown that enhancement of GABAergic signaling impairs olfactory memory in these insects. In fruit flies, for instance, overexpression of the main GABAA receptor gene *resistance to dieldrin* (Rdl) in the anterior paired lateral (APL) neurons, which provide GABAergic input to the MBS, impairs olfactory associative memory acquisition, but not stability. On the contrary, Rdl knockdown in the same neurons enhances memory acquisition but not memory stability (Liu et al., 2007). Rdl overexpression abolishes the normal calcium responses of the MBs to odors while Rdl knockdown increases these responses. Thus, Rdl seems to negatively modulate olfactory associative learning, possibly by gating the input of olfactory information into the MBs (Liu et al., 2007). In honey bees, delivery of the GABAergic agonist muscimol 20 min before conditioning as well as photolytic uncaging of GABA during appetitive olfactory PER conditioning impairs appetitive memory formation (Raccuglia and Mueller, 2013). Photolytic uncaging, which allows for a more precise temporal control of GABA action in the nervous system, showed that the MBs of the honey bee are GABA-sensitive during a specific time period of CS–US pairing during which GABA release impairs associative memory formation (Raccuglia and Mueller, 2013). Interestingly, upon 3-trial PER conditioning, injection of the agonist muscimol 20 in before conditioning did neither affect learning nor 2-h retention but impaired 24-h retention. In our case, the complementary strategy of injecting GABA-receptor antagonists 15 min before a multiple-trial conditioning did also preserve learning (see Figure 1) but, in the case of PTX, it impaired specifically reversal learning when injected into the calyces. Due to differences in protocols (number of trials, reversal learning in our work vs. single-phase, absolute conditioning in Raccuglia and Mueller, 2013), and in procedures, which either enhanced (Raccuglia and Mueller, 2013) or diminished GABAergic signaling (our work), a direct comparison between our results and previous works is difficult. Yet, both have in common that ionotropic GABA signaling plays an important role in olfactory learning and memory.

### GABA Neurotransmission in the Mushroom Bodies of the Honey Bee

We used PTX (Sattelle et al., 1991; Buckingham et al., 2005) and CGP_54626_ (Blankenburg et al., 2015) to target ionotropic and metabotropic GABA receptors, respectively. Both drugs have been shown to block GABAergic neurotransmission in honey bees and affect olfactory processing (PTX: Sachse and Galizia, 2002; Barbara et al., 2005; Boumghar et al., 2012; Froese et al., 2014; CGP_54626_: Dupuis et al., 2010; Froese et al., 2014).

GABA is the main inhibitory neurotransmitter in insects (Hosie et al., 1997) and is largely distributed in the central nervous system (e.g., honey bee: Bicker, 1999; *Drosophila*: Enell et al., 2007; locust: Leitch and Laurent, 1996). The precise function of GABAergic receptors and neurotransmission has been extensively studied, with GABA-immunoreactivity being mainly localized in the optic lobes (Schäfer and Bicker, 1986; Kiya and Kubo, 2010), local interneurons of the AL and extrinsic FNs innervating the MBs (Bicker et al., 1985; Ganeshina and Menzel, 2001; Okada et al., 2007). However, the specific type of GABA receptor mediating GABAergic signaling in the MBs remains unknown. Our results indicate that ionotropic GABA receptors are necessary for reversal learning, while metabotropic GABA receptors are dispensable. Moreover the fact that PTX injections into the calyces, but not into the lobes, impaired reversal learning, not only serves as a powerful control for a targeted localization of the drug applied but also allows referring this result to a specific subset of FNs. Indeed, no feed-forward (i.e., external and afferent) GABAergic signaling to the calyces has been described, and GABAergic feedback in the MBs of the honey bee brain is provided by two tracts of FNs, the A3v cluster of the protocerebro-calycal tract (PCT), which connects vertical and medial lobes with the calyces (Rybak and Menzel, 1993), and the A3d cluster, which provides local feedback from the medial and vertical lobes to the vertical lobe, probably acting on MB extrinsic neurons projecting to premotor neurons (Okada et al., 2007). Our results suggest that from these two tracts, only the A3v cluster is necessary for achieving reversal olfactory learning through ionotropic GABA receptors.

### GABAergic Feedback Neurons as a Modulatory Circuit Facilitating Reversal Learning in Insects

Olfactory information is conveyed to the MBs *via* projection neurons (PNs), which contact KC, the constitutive MB neurons (Kirschner et al., 2006). Kenyon cells also receive value signals from ventral unpaired medial maxillar one neuron (VUMmx1), the neuron encoding sucrose reward in the bee brain (Hammer, 1993), so that they are subject to experience-dependent plasticity as shown by changes in their connectivity following long-term olfactory memory formation (Hourcade et al., 2010). A3v neurons feedback onto the calyces and could thus inhibit the input to KCs; this inhibition may be crucial to decrease their responses to odorants subject to a change in valence as in the transition between phases inherent to reversal learning.

This hypothesis is supported by findings in the fruit fly *Drosophila melanogaster* where the APL neurons are GABAergic and resemble the A3v cluster (Liu and Davis, 2009). Functional disruption of the APL-to-MB signaling using neurogenetic tools impairs visual reversal learning by impeding the specific suppression of the initial memory (Ren et al., 2012). A similar conclusion was reported for olfactory reversal learning in fruit flies (Wu et al., 2012) but this work did not verify whether second-phase olfactory acquisition was possible upon APL inhibition. Using a simple olfactory discrimination instead of reversal learning, Lin et al. (2014) showed that olfactory sparse coding in the MBs is due to the inhibitory feedback of APL neurons. Kenyon cells activate APL neurons and they inhibit in turn KCs. Disrupting the Kenyon cell-APL feedback loop decreases the sparseness of KCs odor responses, increases inter-odor correlations and prevents flies from learning to discriminate similar, but not dissimilar, odors. Thus, GABAergic feedback inhibition to the calyces suppresses KCs activity to maintain sparse, decorrelated odor coding and thus the odor specificity of memories (Lin et al., 2014). While this mechanism may support the learning of each olfactory discrimination and thus solving each of the two consecutive phases of reversal learning, it may be particularly enhanced in the case of ambiguity of stimulus valence upon phase transition. As PCT FNs exhibit experience-dependent plasticity (Haehnel and Menzel, 2010), A3v neurons may enhance their inhibition to the learned stimuli in order to favor the reversal of stimulus valence.

Note that neither retrieval nor extinction were impaired by PTX injection so that ionotropic GABAergic inhibition affected a different computational process related to the capacity of inverting stimulus value. When a subject experiences a reversed CS-US contingency, a dual process occurs: a new excitatory learning of the formerly non-reinforced stimulus and a new inhibitory learning of the original CS-US association. In our experiments, PTX-injection into the calyces impaired inhibitory learning of the previous A-US association but not the excitatory learning of the new B-US association, which occurred in a similar way as in the PBS-injected group (see Figure 3). Concluding that extinction learning is the process specifically targeted by PTX, independently of excitatory learning, is, however, incautious as shown by the extinction experiment (A^+^ vs. B^−^ → A^−^ and B^−^) in which extinction was preserved and similar both in the PTX- and the PBS-injected bees (see Figure 6). In this case, the level of ambiguity of stimulus outcome was only partial as a change in stimulus valence occurred for A but not for B. It thus seems more appropriate to suggest that inhibition of GABAergic ionotopic receptors affected the reversal process in a situation of full reversal (i.e., of full ambiguity of stimulus outcome). This hypothesis implies that the excitatory and inhibitory learning involved in the reversal phase might not be fully independent.

All in all, these results suggest that the inhibitory regulation of KCs activity by GABAergic FNs constitutes a generalized mechanism and has a major role in the resolution of conflicting contingencies, thus supporting the resolution of ambiguous tasks.

### GABAergic Feedback Neurons and MBs are Necessary for Solving Ambiguous Learning Tasks, but are Dispensable for Elementary Discriminations

Previous work using procaine-blockade of MBs Na^+^ and K^+^ channels showed that bee MBs are necessary for olfactory reversal learning but are dispensable for learning two consecutive simple olfactory discriminations (Devaud et al., 2007). Our experiments replicated these findings and identified the GABAergic A3v neurons of the MBs as a likely substrate of this effect. Both works thus coincide in the demonstration that MBs, which are definitely a site for storage and retrieval of elemental olfactory memories (Menzel, 1999; Menzel et al., 2001), may be replaced by other structures for acquiring simple forms of learning. A similar conclusion was reached in other studies (Malun et al., 2002; Komischke et al., 2005) that showed that simple olfactory discriminations were possible despite partial MBs ablation by larval treatment with hydroxyurea. Taken together, these results reveal a principle of redundancy in memory formation in the honey bee brain and point out that structures such as the ALs may be alternate sites for the storage and eventual retrieval of olfactory memories induced by simple learning tasks. Indeed, calcium imaging experiments have shown short-term changes in neural activity for a rewarded odorant following learning of a simple olfactory discrimination (Faber et al., 1999). The long-term, simple absolute conditioning with a single odorant paired with sugar also induces structural changes in ALs (Hourcade et al., 2009). Such redundancy is a main difference between bees and flies, as in *Drosophila* functional MBs have been repeatedly shown to be necessary for olfactory learning and memory, even of simple discrimination tasks (Heisenberg, 2003; Gerber et al., 2004; Krashes et al., 2007; Wang et al., 2008).

Reversal learning exhibits only a transient ambiguity of stimulus outcome that occurs at the transition between the two consecutive conditioning phases. Other learning tasks presenting ambiguity of stimulus outcome to a larger extent may also require MB integrity, and more specifically GABAergic feedback signaling from the lobes to the calyces (Giurfa, 2003, 2007). For instance, the unique capacity of honey bees to solve ambiguous patterning discriminations (e.g., positive patterning: A^−^, B^−^ vs. AB^+^; negative patterning: A^+^, B^+^ vs. AB^−^; Deisig et al., 2001, 2002, 2003) where each stimulus is as often rewarded as non-rewarded, may rely on MBs and GABAergic FN signaling. From this perspective, MBs might be necessary during the acquisition of “complex” tasks, i.e., those requiring the resolution of contradictory CS-US associations. Further experiments combining conditioning protocols such as the negative/positive patterning and MB/feedback-neuron blockade will provide insight into this hypothesis.

## Author contributions

All authors (CB, JMD, GI, MG) substantially contributed to the conception of the work, and the analysis and interpretation of data. All of them also contributed in revising the work critically for important intellectual content, and approving the version to be published. They all agree to be accountable for all aspects of the work in ensuring that questions related to the accuracy or integrity of any part of the work are appropriately investigated and resolved. CB also contributed to the acquisition of data, CB and MG drafted the work.

## Conflict of Interest Statement

The authors declare that the research was conducted in the absence of any commercial or financial relationships that could be construed as a potential conflict of interest.

## Abbreviations

AL: Antennal Lobe
APL: Anterior Paired Lateral neurons
CS: Conditional Stimulus
FN: Feedback Neurons
GABA: Gamma-AminoButyric Acid
KC: Kenyon Cells
LH: Lateral Horn
MBs: Mushroom Bodies
PBS: Phosphate Buffer Saline
PCT: Protocerebro-Calycal Tract
PER: Proboscis Extension Reflex
PNs: Projection Neurons
PTX: Picrotoxin
US: Unconditional Stimulus.
